# Reduced Left Ventricular Ejection Fraction Is a Risk Factor for In-Hospital Mortality in Patients after Percutaneous Coronary Intervention: A Hospital-Based Survey

**DOI:** 10.1155/2018/8753176

**Published:** 2018-12-05

**Authors:** Ziliang Ye, Haili Lu, Lang Li

**Affiliations:** ^1^Department of Cardiology, the First Affiliated Hospital of Guangxi Medical University, Nanning, Guangxi, China; ^2^Department of Orthodontics, the Affiliated Dental Hospital of Guangxi Medical University, Nanning, Guangxi, China

## Abstract

**Background:**

To evaluate whether a reduced left ventricular ejection fraction (LVEF) is a risk factor in patients after percutaneous coronary intervention (PCI).

**Methods:**

A retrospective cohort study from February 2013 to January 2017 was performed, and 1600 patients were included (136 patients with EF <50% and 1464 patients with EF ≥50%); all patients underwent PCI. Revascularization, in-hospital mortality, and in-hospital myocardial infarction (MI) during hospitalization were evaluated.

**Results:**

The mean age of patients with EF <50% was 62.18 ± 10.31 years, while the mean age of patients with EF ≥50% was 60.06 ± 10.89 years (P=0.029). In-hospital mortality of patients with EF ≥50% was significantly lower than that of patients with EF <50% (0.12% vs. 3.68%, P<0.001), while no difference was observed in revascularization and in-hospital MI between the two groups (2.39% vs. 2.20%, P=0.892; 0.415% vs. 1.47%, P=0.093, respectively). In the univariate analysis, no significant difference was found in revascularization and in-hospital MI between the two groups (OR: 1.50, 95% CI: 0.95 to 2.38; OR: 0.28, 95% CI: 0.06 to 1.38, respectively) except for in-hospital mortality (OR: 1.12, 95% CI: 1.05 to 1.27). In multivariate analyses, in-hospital mortality of patients with EF ≥50% was still significantly lower than of patients with EF <50% (OR: 1.15, 95% CI: 1.08 to 1.33). There were no differences in revascularization and in-hospital MI between the two groups (OR: 0.85, 95% CI: 0.44 to 1.63; OR: 0.04, 95% CI: 0.00 to 1.84, respectively).

**Conclusions:**

Reduced LVEF is a risk factor for in-hospital mortality in patients after PCI.

## 1. Introduction

With the change of people's living habits and the acceleration of global population ageing, the incidence of coronary heart disease (CHD) is increasing year by year [1–3]. At present, CHD is the leading cause of death in human beings. Research data show that death due to CHD accounted for 13% in 2010, and the death toll was approximately 7029 300[4, 5]. A computer predictive model revealed that CHD will be the leading cause of death worldwide by 2020 [6, 7]. In the United States, approximately 800000 people suffer from acute myocardial infarction every year, and half of those patients die before they arrive in the hospital [8, 9]. Studies related to China showed that in 2020-2029 years, the prevalence of CHD in China will increase by 69%, while the mortality rate will increase by 68% [10, 11]. The morbidity and mortality of CHD have attracted worldwide attention.

Acute coronary syndrome (ACS), including ST-segment elevation myocardial infarction (STEMI), non–ST-segment elevation myocardial infarction (NSTEMI), and unstable angina (UA)[12, 13], is a group of clinical syndromes caused by rupture of coronary atherosclerotic plaques and secondary thrombosis. Its features include sudden onset, severe symptoms, and the state of the illness change rapidness, which should be treated immediately. Studies have shown that after the onset of ACS, timely opening of the obstructed vessels can significantly improve myocardial ischemia reperfusion, left ventricular function, and infarct size and reduce mortality and complications (such as ventricular tachycardia and heart failure) [14]. At present, percutaneous coronary intervention (PCI) is one of the effective methods for timely opening of obstructed blood vessels, thus reducing mortality and improving quality of life [15, 16].

However, many factors also affect the prognosis of patients after PCI. Previous studies have found that atrial fibrillation (AF) is independently associated with mortality after PCI for chronic total occlusions, and AF can increase mortality in 62% (HR 1.62, 95% CI: 1.06–2.47, p = 0.03) [17]. In addition, a prospective cohort study, including 12,347 consecutive patients (1,575 with and 10,772 without diabetes), found that the all-cause mortality rate in diabetic patients over 2 years was significantly higher than that in nondiabetic patients (adjusted RR 1.91, 95% CI: 1.63 to 2.23; p <0.001); the incidence of revascularization in diabetic patients was also significantly higher than that in nondiabetic patients (adjusted RR 1.28, 95% CI: 1.10 to 1.49; p <0.001) [18, 19]. Furthermore, some scholars also found that obesity was associated with a higher risk of target lesion revascularization (HR: 1.39; 95% CI: 1.06 to 1.83; P =0.019) by examining 6,083 patients undergoing PCI with drug-eluting stents [20].

Although the mortality rate of ACS is decreasing, the incidence of heart failure is increasing year by year. Many studies have shown that left ventricular ejection fraction (LVEF) is closely related to the prognosis of ACS patients. Similarly, previous studies have indicated that decreased EF is a risk factor for adverse events during hospitalization and long-term outcomes in patients undergoing PCI. A prospective cohort study [19], including 2,030 patients, found that the mortality rates of patients with low ejection fractions in 30 days (HR: 9.81, 95% CI: 5.23 to 18.42, p <0.0001) and 3 years (HR: 5.03, 95% CI: 3.37 to 7.50, p <0.0001) were significantly higher than of patients with normal ejection fraction. Similarly, Sardi G [21] also found that a decreased LVEF increases the risk of stent thrombosis, which may affect the prognosis of the patients undergoing PCI. However, whether a reduced left ventricular EF can affect the prognosis of the Chinese patients after PCI remains unclear. Therefore, we performed this hospital-based survey to evaluate whether a reduced LVEF is a risk factor in patients undergoing PCI.

## 2. Methods

### 2.1. Study Design

**Figure 1:**
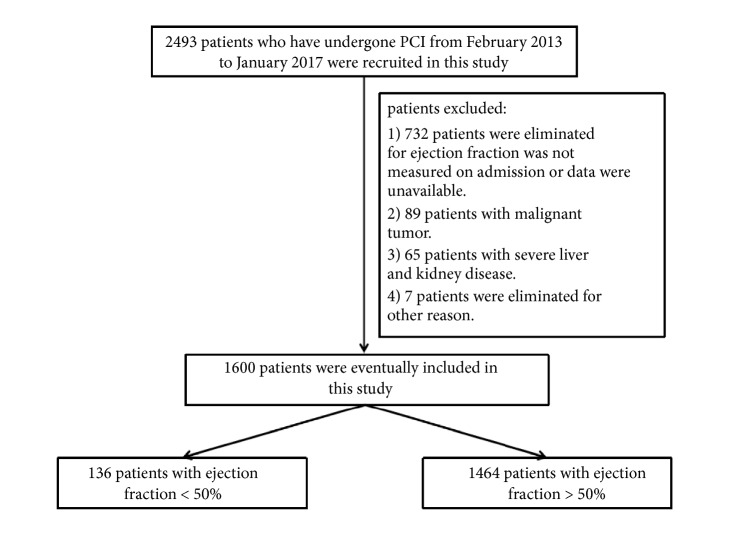
The flow chart of this study.

This study was a retrospective cohort study from February 2013 to January 2017 conducted in the First Affiliated Hospital of Guangxi Medical University, Guangxi, China. 2493 patients who have undergone PCI were recruited in this study. 893 patients were excluded from this study for the following reasons: 732 patients were eliminated for ejection fraction was not measured on admission or data were unavailable, 89 patients were with malignant tumor, 65 patients were with severe liver and kidney disease, and 7 patients were eliminated for other reasons. Finally, 1600 patients were eventually included in this study (136 patients with ejection fraction < 50% and 1464 patients with ejection fraction ≥50%). The flow chart is shown in Figure 1. We use electronic medical record databases of the First Affiliated Hospital of Guangxi Medical University to collect demographics characteristics, comorbidities, and cardiac medications of all patients. The patient's information mainly includes age, sex, body mass index (BMI), systolic blood pressure (SBP), diastolic blood pressure (DBP), heart rate, laboratory examination results, and previous history. This study has been approved by the ethics committee of the First Affiliated Hospital of Guangxi Medical University. Because this study was a retrospective cohort study, and all patients were anonymous, thus the written informed consent was not required. This study was conducted in accordance with the tenets of the Declaration of Helsinki. The data used to support the findings for this study are available from the corresponding author upon request.

Inclusion criteria were as follows: (1) patient who underwent PCI from February 2013 to January 2017 conducted in the First Affiliated Hospital of Guangxi Medical University, Guangxi, China; (2) age more than 18 years old, but less than 75 years old. Exclusion criteria were as follows: (1) patient with malignant tumors, such as colorectal cancer, esophageal cancer, gastric cancer, or liver cancer; (3) renal function severely impaired (estimated glomerular filtration rate <30 ml/min/1.73 m^2^ or dialysis); (4) female patients with pregnancy or suckling period; and (5) data not available during hospitalization after PCI.

### 2.2. Treatment and Procedure

The drugs before and after PCI were given according to accepted guidelines and established practice standards, including aspirin, clopidogrel, and statins. The procedures of PCI and perioperative anticoagulant therapy are carried out in accordance with the accepted guidelines. The use of predilation, intravascular ultrasound, and intraaortic balloon pumps and the type of stent (drug eluting and bare metal) is determined by experts in interventional cardiology. Baseline echocardiography evaluations were performed at admission, and LVEF was measured using the M-mode or modified Simpson's method, as recommended by the American Society of Echocardiography.

### 2.3. Outcomes and Definitions

Revascularization, in-hospital mortality, and in-hospital myocardial infarction (MI) during hospitalization were evaluated through an electronic medical record system of the First Affiliated Hospital of Guangxi Medical University, Guangxi, China. If necessary, an office visit or telephone contact was conducted to confirm the clinical outcome of patients. Revascularization was defined as treatment for recurrent angina in the presence of signs or symptom of myocardial ischemia during hospitalization, including target lesion revascularization (TLR), target vessel revascularization (TVR), nontarget vessel revascularization (non-TVR), or coronary artery bypass graft (CABG). In-hospital mortality included any death during hospitalization, including target MI, stroke, heart failure, ventricular tachycardia, or sudden death. In-hospital MI was established mainly according to the generalized definition of myocardial infarction as a transient increase of laboratory markers specific of myocardial necrosis (CK-MB, or troponin T) in combination with ischemic symptoms and/or typical ECG signs (development of pathologic Q-waves or ST-segment elevation or depression).

### 2.4. Statistical Analysis

In this study, 1 600 patients were divided into two groups based on left ventricular ejection fraction (136 patients with EF <50% and 1464 patients with EF ≥50%). Results are presented as the number (percent) for categorical variables or mean ± standard deviation (SD) for continuous variable. Categorical variables were compared using the Chi square test, and continuous variables between the two groups were compared using unpaired Student's t-test. In this study, we used odds ratio (OR) and 95% confidence interval (CI) to estimate the results. In addition, we also performed univariate and multivariate logistic regression analyses to estimate the results between the two groups. In multivariate logistic regression analyses, demographic and clinical factors, including age, BMI, SBP, DBP, heart rate, creatinine, uric acid, bilirubin, total cholesterol, triglyceride, high-density lipoprotein (HDL), low-density lipoprotein (LDL), sex, complications, medication history, and diet history, were adjusted to obtain accurate results.

All reported probability values were 2-sided, and a* P* value<0.05 was considered statistically significant. All statistical analyses were performed using SPSS version 22 for Windows (Armonk, New York, USA) and GraphPad Prism 5 (GraphPad Software, La Jolla, CA).

## 3. Results

### 3.1. Patient Characteristics

An analysis of the clinical characteristics of study subjects revealed that systolic blood pressure (SBP), diastolic blood pressure (DBP), heart rate, uric acid, total bilirubin, total cholesterol, low-density lipoprotein, sex, atrial fibrillation (AF), history of stroke, history of PCI, history of CABG, diabetes, smoking, aspirin, *β*-blocker, angiotensin-converting enzyme inhibitors (ACEIs), and statin were not significantly different between two groups (Table 1). However, age, body mass index (BMI), serum creatinine, triglyceride, high-density lipoprotein, history of heart failure, hypertension, clopidogrel, and calcium antagonists were significantly different between the two groups (Table 1). A reduced LVEF was likely to be associated with high age, low BMI, high serum creatinine, low triglyceride, low high-density lipoprotein, high history of heart failure, low history of hypertension, high use of clopidogrel, and low use of calcium antagonists.

**Figure 2:**
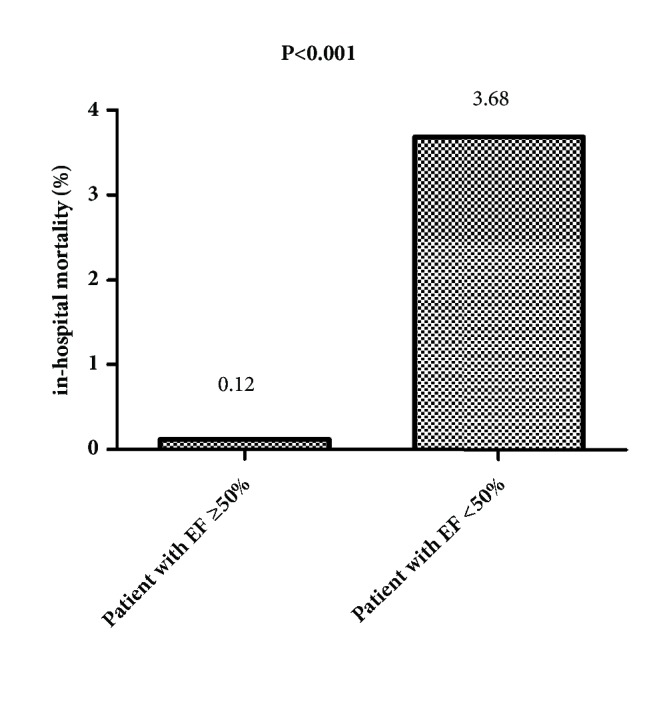
The in-hospital mortality of patients with EF ≥50% and patients with EF <50% (0.12% vs. 3.68%, P<0.001).

**Figure 3:**
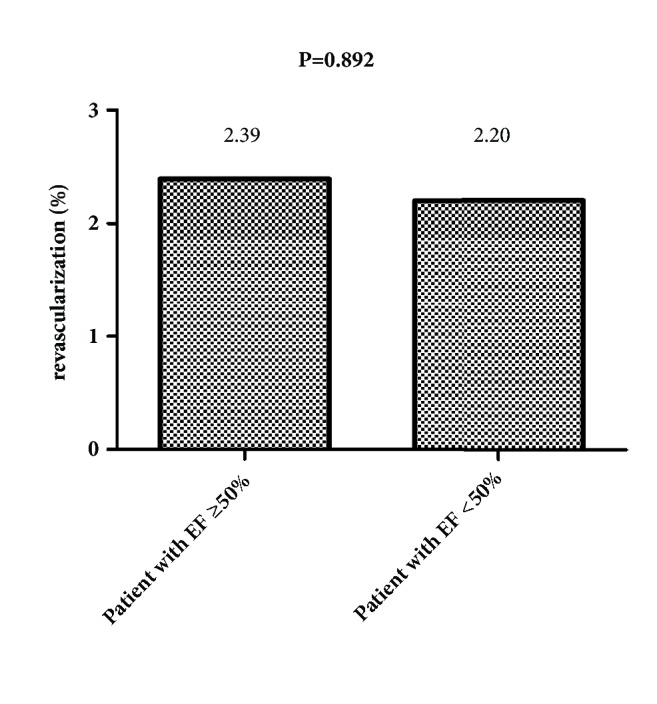
The revascularization rate of patients with EF ≥50% and patients with EF <50% (2.39% vs. 2.20%, P=0.892).

**Figure 4:**
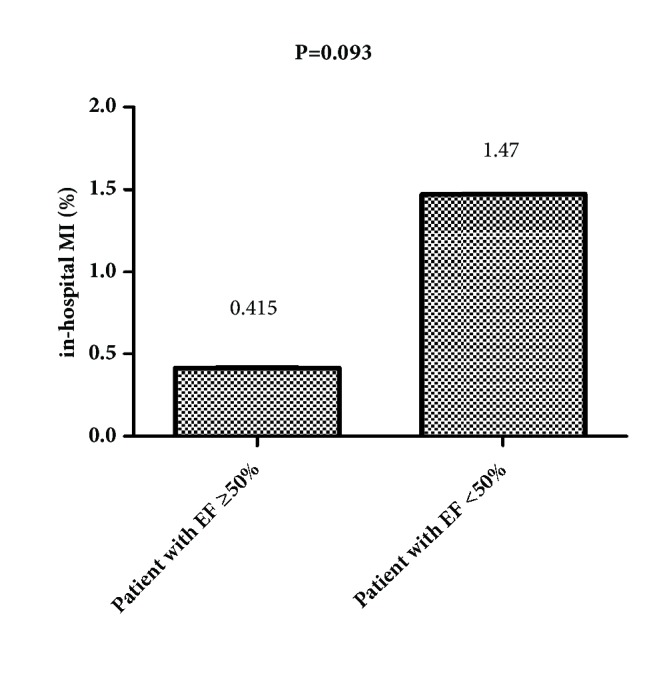
The in-hospital MI rate of patients with EF ≥50% and patients with EF <50% (0.415% vs. 1.47%, P=0.093).

The in-hospital mortality of patients with EF ≥50% was significantly lower than of patients with EF <50% (0.12% vs. 3.68%, P<0.001) (Figure 2), while no difference was observed in revascularization and in-hospital MI between the two groups (2.39% vs. 2.20%, P=0.892; 0.415% vs. 1.47%, P=0.093, respectively) (Figures 3 and 4).

In univariate analysis, no significant difference was found in revascularization and in-hospital MI between the two groups (OR: 1.50, 95% CI: 0.95 to 2.38; OR: 0.28, 95% CI: 0.06 to 1.38, respectively), while the in-hospital mortality of patients with EF ≥50% was significantly lower than of patients with EF <50% (OR: 1.12, 95% CI: 1.05 to 1.27) (Table 2). In multivariate logistic regression analyses, the in-hospital mortality of patients with EF ≥50% was still significantly lower than of patients with EF <50% (OR: 1.15, 95% CI: 1.08 to 1.33). However, there were no differences in revascularization and in-hospital MI between the two groups after adjusting for demographic and clinical factors (OR: 0.85, 95% CI: 0.44 to 1.63; OR: 0.04, 95% CI: 0.00 to 1.84, respectively) (Table 2).

## 4. Discussion

In the present study, we aim to evaluate whether a reduced LVEF is a risk factor in patients after PCI. We found that patients with low EF had a higher in-hospital mortality than patients with normal EF (P<0.05). Similar results were obtained when potential confounding factors were adjusted using univariate and multivariate logistic regression analyses (P<0.05). However, no significant association was observed between a reduced LVEF and revascularization and in-hospital MI (all P >0.05). To the best of our knowledge, this retrospective cohort study, drawn from a cohort of 1600 patients, is the largest study on Chinese's population to evaluate the clinical outcome of patients with a reduced LVEF after PCI.

Consistent with previous studies, our study also found a significant negative correlation between LVEF and in-hospital mortality in patients after PCI. It means that patients with low EF are more likely to die at the hospital. After adjusting for potential confounding factors, the in-hospital mortality of patients with EF <50% will increase by 15% (OR: 1.15, 95% CI: 1.08 to 1.33), compared to patients with EF ≥50%. To date, several studies have observed the association between LVEF and clinical outcome of patients after PCI. A prospective registry conducted in Iran [19], including 293 patients with EF ≤40%, 268 patients with EF ranged from 41 to 49%, and 1 469 patients with EF ≥50%, found that patients with left ventricular dysfunction or a reduced LVEF had higher major adverse cardiac events (HR: 2.07, 95% CI: 1.03 to 4.16) and cardiac death rates (HR: 5.49, 95% CI: 1.29 to 23.3), compared to patients with EF ≥50%. In addition, another prospective cohort study [22], including 304 patients who had undergone primary PCI, was performed to evaluate the association between LVEF and in-hospital outcomes of patients with acute ST-segment elevation myocardial infarction (STEMI), and the result indicated that a reduced LVEF is associated with a higher incidence of in-hospital adverse events (P <0.05). Furthermore, a recent study also pointed out that decreased LVEF will increase the risk of stent thrombosis [21].

It is well known that LVEF can be used as an indicator of cardiac function and has been widely used in routine clinical practice [23–25]. Some previous studies have shown that the decline in EF is a risk factor for many diseases. For example, an individual patient data meta-analysis [26], including 10 347 patients with preserved left ventricular ejection fraction (HF-PEF) and 31 625 patients with heart failure and reduced EF (HF-REF), indicated that patients with HF-REF have a significant increase in death (P<0.05). Moreover, similar results were obtained after subgroup analysis was performed (all P<0.05). In addition, a prospective study found that the cardiovascular and HF readmission rates were higher in patients with a reduced left ventricular EF when compared with patients with a normal EF (HR: 1.335 [95% CI: 1.288 to 1.383], p < 0.0001; HR: 1.162 [95% CI: 1.098 to 1.229], p <0.0001, respectively) [19]. In contrast, a study conducted by Kobayashi Y et al. [27] found that there is no significant association between EF and fractional flow reserve value. It is important to note that in the study performed by Kobayashi Y et al., the index of observation is fractional flow reserve value, and patients did not have a PCI operation, which may explain the inconsistency of different results.

Our study has several strengths that need to be pointed out as follows. First, compared to previous studies, 1 600 patients were included in this study. Thus, we had the statistical power to assess this important association between LVEF and clinical outcome in patients after PCI. Second, although our study is a retrospective cohort study in nature, we performed multivariate logistic regression analyses minimizing residual confounding, including adjustment for demographic and clinical factors. Third, our study is a single-center study, not a multicenter study. Therefore, some potential measurement bias, such as variations in formulary restrictions and differences among the database structures, can be reduced to a minimum. Fourth, researchers for this study did not change, thus the observation bias and the follow-up bias can be reduced to a minimum and obtain a credible result.

Our study also showed several limitations. First, as mentioned above, our study is a retrospective cohort study, and we cannot obtain the causal link between LVEF and in-hospital mortality. Second, some diabetes patients who were treated with thiazolidinediones, which can increase the risk of hospitalization for heart failure [28], were included in this study. However, sensitivity analysis cannot be conducted without much data on thiazolidinediones. Third, as a limitation of retrospective study, some previous baseline data are not available. Similarly, our study also did not acquire part of the patient's information. For example, the degree of stenosis of coronary artery disease, type of stent implantation, and the number of implanted stents were not available in this study. Fourth, the study object was based upon the Chinese population, and the results were not necessarily applicable to other populations.

## 5. Conclusion

In this retrospective analysis, we found reduced LVEF is a risk factor for in-hospital mortality in patients after PCI. However, more studies are needed to confirm this conclusion.

## Figures and Tables

**Table 1:** Baseline clinical characteristics of patients stratified by ejection fraction (at admission).

Variables	Patients with EF <50%	Patients with EF ≥50%	*P* value
N	136	1464	

Age (year)	62.18 ± 10.31	60.06 ± 10.89	0.029

BMI (kg/m^2^)	22.99 ± 3.14	23.95 ± 3.83	0.023

SBP (mmHg)	98.83 ± 28.82	102.55 ± 28.69	0.150

DBP (mmHg)	77.93 ± 11.30	77.12 ± 11.78	0.440

Heart rate (times/ min)	71.55 ± 13.02	72.12 ± 11.13	0.581

serum creatinine (umol/L)	83.17 ± 68.07	71.49 ± 30.89	<0.001

Uric acid (mmol/L)	316.92 ± 106.69	302.22 ± 91.59	0.086

Total bilirubin (*μ*mol/L)	11.03 ± 6.16	9.61 ± 8.26	0.055

Total cholesterol (mmol/L)	4.10 ± 1.00	4.27 ± 1.06	0.086

Triglyceride (mmol/L)	1.57 ± 0.80	1.95 ± 1.46	0.005

High density lipoprotein (mmol/L)	1.01 ± 0.31	1.08 ± 0.31	0.022

Low density lipoprotein (mmol/L)	2.61 ± 0.88	2.65 ± 0.93	0.657

Sex			0.628

female	41 (30.15%)	471 (32.17%)	

male	95 (69.85%)	993 (67.83%)	

History of heart failure			<0.001

no	95 (69.85%)	1290 (88.24%)	

yes	41 (30.15%)	172 (11.76%)	

History of atrial fibrillation			0.076

no	130 (95.59%)	1434 (97.95%)	

yes	6 (4.41%)	30 (2.05%)	

History of stroke			0.051

no	124 (91.18%)	1392 (95.08%)	

yes	12 (8.82%)	72 (4.92%)	

History of PCI			0.806

no	128 (94.12%)	1370 (93.58%)	

yes	8 (5.88%)	94 (6.42%)	

History of CABG			0.778

no	135 (99.26%)	1456 (99.45%)	

yes	1 (0.74%)	8 (0.55%)	

Hypertension			<0.001

no	87 (63.97%)	694 (47.44%)	

yes	49 (36.03%)	769 (52.56%)	

Diabetes			0.615

no	104 (76.47%)	1146 (78.33%)	

yes	32 (23.53%)	317 (21.67%)	

Smoking			0.529

no	86 (63.24%)	965 (65.92%)	

yes	50 (36.76%)	499 (34.08%)	

Types of patients			0.945

STEMI	51 (37.5%)	536 (36.6%)	

NSTEMI	42 (30.8%)	443 (30.2%)	

Unstable angina	43 (31.7%)	485 (33.2%)	

Aspirin			0.571

no	1 (0.74%)	19 (1.30%)	

yes	135 (99.26%)	1443 (98.70%)	

Clopidogrel			0.001

no	5 (3.68%)	65 (4.44%)	

yes	129 (94.85%)	1397 (95.49%)	

No clear	2 (1.47%)	1 (0.07%)	

*β*-blocker			0.816

no	37 (27.21%)	412 (28.14%)	

yes	99 (72.79%)	1052 (71.86%)	

ACEI			0.344

no	53 (38.97%)	632 (43.17%)	

yes	83 (61.03%)	832 (56.83%)	

Calcium antagonists			<0.001

no	122 (89.71%)	1077 (73.57%)	

yes	14 (10.29%)	387 (26.43%)	

Statin			0.777

no	9 (6.62%)	88 (6.01%)	

yes	127 (93.38%)	1376 (93.99%)	

Data are presented as number (percent) or mean ±standard deviation.

Body mass index=BMI; systolic blood pressure=SBP; diastolic blood pressure=DBP; percutaneous coronary intervention=PCI; coronary artery bypass grafting=CABG; ST segment elevation myocardial infarction=STEMI; non-ST-elevation myocardial infarction=NSTEMI; ACEIs=angiotensin-converting enzyme inhibitors.

**Table 2:** Univariate and multivariate logistic regression analyses.

	Unadjusted		Adjusted*∗*	
Variables	OR (95% CI)	*P* value	OR (95% CI)	*P* value

Revascularization	1.50 (0.95, 2.38)	0.0794	0.85 (0.44, 1.63)	0.6269

In-hospital mortality	1.12 (1.05, 1.27)	0.0130	1.15 (1.08, 1.33)	0.0112

In-hospital MI	0.28 (0.06, 1.38)	0.1168	0.04 (0.00, 1.84)	0.1008

*∗*Adjusted for demographic and clinical factors. Odds ratio=OR; myocardial infarction=MI; confidence interval=CI.

Results are presented as OR (95% CI).

## Data Availability

The data used to support the findings of this study are available from the corresponding author upon request.
